# Maternal Weight Variation in Different Intrauterine Environments: An Important Role of Hypertension

**DOI:** 10.1055/s-0039-1683373

**Published:** 2019-04-02

**Authors:** Bianca da Rosa Cazarotto, Charles Francisco Ferreira, Amanda Pereira Ferreira, Luciano Santos Pinto Guimarães, Vera Lúcia Bosa, Juliana Rombaldi Bernardi, Marcelo Zubaran Goldani, Clécio Homrich da Silva

**Affiliations:** 1 Postgraduate Program in Child and Adolescent Health, Universidade Federal do Rio Grande do Sul, Porto Alegre, RS, Brazil

**Keywords:** body mass index, body weight, hypertension, postpartum period, índice de massa corporal, peso corporal, hipertensão, período pós-parto

## Abstract

**Objective** Different intrauterine environments may influence the maternal prepregnancy body weight (BW) variation up to 6 months postpartum. The objective of the present study was to verify the association of sociodemographic, obstetric, nutritional, and behavioral factors with weight variation in women divided into four groups: hypertensive (HM), diabetic (DM), smokers (SM), and control mothers (CM).

**Methods** It was a convenience sample of 124 postpartum women recruited from 3 public hospitals in the city of Porto Alegre, state of Rio Grande do Sul, Brazil, between 2011 and 2016. Multiple linear regressions and generalized estimating equations (GEE) were conducted to identify the factors associated with maternal weight variation. For all GEE, the maternal weight measurements were adjusted for maternal height, parity, educational level, and the type of delivery, and 3 weight measurements (prepregnancy, preceding delivery, and 15 days postpartum) were fixed.

**Results** A hierarchical model closely associated the maternal diagnosis of hypertension and a prepregnancy body mass index (BMI) classified as overweight with maternal weight gain measured up to the 6^th^ month postpartum (the difference between the maternal weight at 6 months postpartum and the prepregnancy weight). These results showed that the BW of the HM group and of overweight women increased ∼ 5.2 kg 6 months postpartum, compared with the other groups. Additionally, women classified as overweight had a greater BW variation of 3.150 kg.

**Conclusion** This evidence supports the need for specific nutritional guidelines for gestational hypertensive disorders, as well as great public attention for overweight women in the fertile age.

## Abstract (pt)

**Resumo:**

**Objetivo** Diferentes ambientes intrauterinos podem influenciar a variação de peso corporal pré-gestacional materno até 6 meses pós-parto. O objetivo do presente estudo foi verificar a associação de fatores sociodemográficos, obstétricos, nutricionais e comportamentais com a variação de peso em mulheres divididas em quatro grupos: hipertensas (HM), diabéticas (DM), tabagistas (SM) e controles (CM).

**Métodos** Amostra de conveniência de 124 puérperas recrutadas em 3 hospitais públicos da cidade de Porto Alegre, Rio Grande do Sul, Brasil, entre 2011 e 2016. Regressões lineares múltiplas e modelos de equações de estimativas generalizadas (GEE) foram realizados para identificar os fatores associados à variação do peso materno. Para todas as GEE, as medidas de peso materno foram ajustadas para a estatura materna, paridade, escolaridade e tipo de parto, e três medidas de peso (pré-gravidez, anterior ao parto e 15 dias pós-parto) foram fixadas.

**Resultados** Um modelo hierárquico associou o diagnóstico materno de hipertensão e o índice de massa corporal (IMC) pré-gestacional de sobrepeso com ganho de peso materno medido até o 6° mês pós-parto (diferença entre o peso materno aos 6 meses pós-parto e o peso pré-gestacional). Estes resultados mostraram que o grupo HM e mulheres com sobrepeso aumentaram o peso corporal em ∼ 5,2 kg 6 meses pós-parto, em comparação com os demais grupos. Além disso, as mulheres classificadas com sobrepeso tiveram uma variação maior de peso corporal, de 3,150 kg.

**Conclusão** Evidenciou-se a necessidade de diretrizes nutricionais específicas para distúrbios hipertensivos gestacionais, bem como de maior atenção dos serviços de saúde públicos para mulheres com excesso de peso em idade fértil.

## Introduction

Epidemiological reported the influence of certain environmental factors, in the beginning of life, with changes in the genetic load expression of the subject, determining a pattern of health and disease in a peculiar form during lifetime.^1^ Likewise, preclinical and clinical data point in the same direction, suggesting a strong association between adverse environments in the fetal life and/or in the postnatal life and the emergence of non-communicable lifelong chronic diseases^2^ and the inference that developmental and fetal growth conditions determine the metabolic adjustments involved in particular outcomes.^3^

Following this rationale, Barker et al^4^ proposed the hypothesis that adverse intrauterine conditions increased the risk of developing cardiovascular diseases in adulthood, and demonstrated that low birthweight newborns remained persistently biologically different from those with proper weight until adulthood. Supported in the Developmental Origins of Health and Disease (DOHaD) approach by Barker, adverse intrauterine environments and the early plasticity of postnatal life are linked to alterations in the metabolic programming during the child development, which may lead to the expression of different phenotypes of diseases in adulthood, such as obesity, hypertension, and diabetes mellitus.^5^

While constituting an important fact in the association between early life and its risk for diseases (due to an adverse intrauterine environment), few studies explored the maternal characteristics involved in this complex cycle (gestation-puerperium). An appropriate gestational weight gain, for example, displays a direct relation to several outcomes in maternal and child health.^6^ The prepregnancy maternal weight is important to trace the direction of gestational weight gain, since an insufficient weight gain is related to preterm birth, to low birthweight, or to small newborn for the gestational age, as well as being a risk factor for late onset of breastfeeding.^7^ On the other hand, excessive maternal weight gain demonstrated an association with the development of gestational diabetes mellitus, of preterm birth, of cesarean section, of large newborn for the gestational age, of postpartum maternal weight retention and, consequently, of maternal and child overweight and obesity.^8^

In this perspective, the present study aims at understanding some maternal influences in order to contribute to the knowledge of how prepregnancy, gestational, and postpartum maternal weight variation may be modulated by maternal stress perception, depressive symptoms, physical activity, and feeding behavior. In this sense, parameters such as maternal age, educational level, previous gestation, breastfeeding practice, antenatal care visits, maternal ethnicity, family income, planning of gestation, type of delivery, and marital status were also evaluated.

## Methods

This is an observational, longitudinal study, with a sample composed of pairs of mothers and newborns (*n *= 400) recruited in 2 public hospitals of Porto Alegre, state of Rio Grande do Sul, Brazil, consisting in an observational cohort of different intrauterine environments.^9^ These mothers resided in Porto Alegre and were invited to participate in the present study. After signing the consent form, they were included and interviewed at the hospitals within between 24 and 48 hours postpartum. The sample excluded HIV-positive mothers, and preterm newborns, twins or more, who had congenital diseases or required hospitalization. The present research was approved by the Ethics and Research Committees of the Hospital das Clínicas de Porto Alegre (HCPA, in the Portuguese acronym) and of the Grupo Hospitalar Conceição (GHC, in the Portuguese acronym) (numbers 11.0097 and 11.027, respectively).

The sample was divided into four intrauterine environment groups, according to the fetal exposure conditions during pregnancy, considering the following criteria:

- Diabetic mothers (DM) – who reported the diagnosis of diabetes mellitus (types 1 and 2) and gestational diabetes;
- Hypertensive mothers (HM) – who reported the diagnosis of hypertensive disorders (preeclampsia, eclampsia, preeclampsia superimposed on chronic hypertension, chronic hypertension, or gestational hypertension);
- Smoking mothers (SM) – who answered positively to the question regarding smoking during pregnancy, regardless of the frequency and the number of cigarettes.
- Control mothers (CM) – who, along with the newborns, did not present any of the aforementioned conditions.

### Sample Size

The sample size was estimated for a large Cohen effect considering 2 groups (Cohen’s d = 0.8), resulting in 2 control subjects for each intrauterine environment subject. Thus, 20 participants and 40 controls were estimated for each environment, this size being sufficient to detect the difference in the variation in the maternal body weight 6 months postaprtum, according to Zanotti et al.^10^ All of the sample calculations were performed in WinPepi for Windows, version 11.44.

## Study Design

The recruitment of the participants was performed in the HCPA, in the Hospital Nossa Senhora da Conceição, and in the Fêmina Hospital (the last two belonging to the GHC). All of the hospitals serve the Brazilian public health system (SUS, in the Portuguese acronym) and have similar characteristics of maternal and childcare. After the recruitment and the 1^st^ interview, the postnatal follow-up was performed on 5 occasions: on the 7^th^ and 15^th^ days, and on the 1^st^, 3^rd^, and 6^th^ months. Three of these interviews were home visits (at 7 and 15 days, and at 3 months) and the other 2 were scheduled at the Clinical Research Center (CPC, in the Portuguese acronym) of the HCPA. Clinical supervisors and interviewers have been trained and certified by the researchers who coordinated the present study, for the standardizing of the data collection.

### Studied Factors

Postpartum interview: a structured questionnaire collected sociodemographic (that is, age, ethnicity, educational level, marital status, occupation, type of delivery, number of medical appointments during the pregnancy), and economic (family income in Brazilian reais [BRL], the currency in Brazil) information, and the International Physical Activity Questionnaire—Short Form (IPAQ-SF) was also applied.

Interview at 7 days postpartum: The Food Frequency Questionnaire (FFQ) was applied in order to estimate retrospectively the total calorie intake during the gestation. This instrument showed 8 consumption frequency options (ranging from “more than 3 times per day” to “never or almost never”) of 96 items, with standardized portions of homemade measures or units that evaluated their consumed quantities.

Interview at 1 month postpartum: the Perceived Stress Scale (PSS 14), translated and validated for the Portuguese language in Brazil, was used.^11^ It consists of 14 items with response options ranging from 0 to 4 (0 = never, 1 = almost never, 2 = sometimes, 3 = often, 4 = always). The sum of the answers provided scores ranging from 0 (no stress) to 56 (extreme stress).

Interviews at 1, 3, and 6 months postpartum: The Edinburgh Postpartum Depression Scale (EPDS) was applied. The EPDS is a self-registration instrument composed of 10 items regarding the previous 7 days, whose options are scored (from 0 to 3) according to the presence of the intensity of the symptom, such as depressive mood (feeling of sadness, self-deprecation and guilt, death or suicidal thoughts), loss of pleasure in activities previously considered pleasant, fatigue, decreased ability to think, to concentrate or to make decisions, and physiological symptoms (insomnia or hypersomnia), and changes in behavior (crying spells). The maximum total sum of the scores of the answers is 30, with values ≥ 12 being considered as depressive symptomatology, as defined in the validation of the scale for Brazil.^12^

Moreover, the analyzed data included maternal weight (in kilograms) and height (in centimeters) during prepregnancy and during the postpartum interviews (height: preceding delivery, at the 1^st^ and 7^th^ months postpartum; weight: prepregnancy, preceding the delivery, at the15^th^ day postpartum, and at the 1^st^, 3^rd^, and 6^th^ months postpartum). The maternal weight was measured using a digital scale (Marte, LC200 PP, Plenna Balanças, Bom Retiro, SP, Brazil) with a maximum capacity of 150 kg and an accuracy of 100 g, placed on a flat surface. The weight was measured with the mother in the upright position, barefoot, and wearing light clothes. The height was measured with a professional anthropometer (Alturaexata, Sanny, São Bernardo do Campo, SP, Brazil) fixed to the wall at 90 degrees from the floor. The body mass index (BMI, in kg/m^2^) was calculated in each period (during prepregnancy, in each gestational trimester, and in the postpartum analyzes).

Besides, the breastfeeding duration was assessed in all of the interviews. The issues related to breastfeeding and complementary meals were collected.

### Statistical Analyses

Regarding the data processing, the database double entry and review were performed using PASW Statistics for Windows, Version 18.0 (SPSS Inc., Chicago, IL, USA).

Continuous variables were expressed as mean and standard deviation (SD) or median and interquartile range, defined by the Shapiro-Wilk test. Categorical variables were described by absolute and relative frequencies. A one-way analysis of variance (ANOVA) with the Tukey post hoc test, or the Kruskal-Wallis test with the Dunn post hoc test were applied to compare the means between continuous variables. On the other hand, categorical variables were submitted to an intragroup comparison using the Chi-squared test with standardized adjusted residual analysis.

Both univariate and multiple linear regression analyses were performed, with maternal weight change (the difference between the maternal weight at 6 months postpartum and the prepregnancy weight), in kilograms, as the dependent variable, and maternal skin color, maternal age, pregnancy planning, household income, marital status, number of antenatal care visits, stress perception, depressive symptoms in the first month postpartum, lactation practice, type of delivery, food consumption guidance during gestation, educational level, prepregnancy BMI, calorie intake during gestation, physical activity during gestation, and the four intrauterine environment groups as independent variables.

Although the maternal weight was measured in all of the interviews, the maternal weight variation was entered in all of the regression models in order to control for variation in the timing of measurement. A reduction model (backward elimination) method was applied with a significance level of 5% in order to produce an inclusive, reduced model. For this, all of the variables were placed in the model, and the variables less associated with the outcome (that is, the ones with the highest *p*-value) were excluded. The process was performed until only significant variables remained in the final model. In addition, an analysis of model fit was conducted, and the reduction model procedure was reapplied. Subjects with missing information regarding factors included in the models were excluded from the analysis. All of the two-way interactions were tested. A hierarchical model, defined by the authors regarding the distal and proximal variables, was produced by multiple logistic regressions in four blocks. As a summary of the proportion of maternal weight variation explained by the model, the final regression unstandardized coefficient (B) was calculated.

GEE considers time measurement as a factor, and it was assessed how the maternal weight underwent modifications among the groups (different intrauterine environments), the moments (time measurements), and the interaction of both factors (groups and moments) were analyzed in generalized linear models (GLMs). A covariance matrix with a robust estimator, a working exchangeable correlation matrix, and a normal distribution with identity binding function was used. For all of the GLMs, the maternal weight measurements were adjusted for some variables (e.g., maternal height, parity, maternal educational level, and type of delivery), and measurements of prepregnancy, before the delivery, and 15 days postpartum maternal weights were requested. The Bonferroni post hoc test was performed when the GLM was significant. The significance level addopted for all analysis was set at 5%, except for the modeled interactions (likelihood method), since it was set at 10%, due to the low power of the tests to show them.

## Results

Out of a total of 400 pairs of mothers and newborns, 124 displayed all of the variable information required as fixed factors (such as prepregnancy, preceding delivery, and 15-day postpartum weight) for the GLM analysis. Therefore, the final sample consisted of 124 women, with a median age of 29.0 years old (95% confidence interval [CI]: 24.0–34.0 years old). The median prepregnancy weight was 64.5 kg (95% CI: 56.5–78.0 kg), and the median prepregnancy BMI was 25.3 kg/m^2^ (95% CI: 21.7–28.8 kg/m^2^). Most of the participants had studied for > 8 years (79.8%), were married or living with a partner (88.7%), white (63.7%), and multiparous (62.1%). Among the 82 women who had previous children, a further analysis of parity revealed that 42 participants (51.2%) had < 3 children, and that 40 (48.8%) had ≥ 3 children. Regarding socioeconomic status, 109 (87.9%) women had a household income of 1 Brazilian minimum wage (Brazilian household income unit reference for 2017: BRL 937.00). Most of the participants delivered by vaginal birth (62.9%), and were nonsmokers (nonsmokers = 49.2%, ex-smokers = 44.4%, smokers = 6.5%).

The analysis of the groups revealed that antenatal care visits were different among groups (one-way ANOVA, F [3.120] = 5.716; *p* = 0.001], since the DM and the HM groups had more antenatal care visits when compared with the SM group (Tukey post hoc test, *p* ≤ 0.05). Additionally, most of the participants of the HM group were classified as presenting with gestational hypertension (27.8%) or preeclampsia (*n* = 6, 35.3%), chronic hypertension (*n* = 4, 23.5%), eclampsia (*n* = 1, 5.9%), preeclampsia syperimposed to chronic hypertension (*n* = 2, 11.8%), and gestational hypertension (*n* = 4, 23.5%). Regarding prepregnancy maternal weight (Kruskal-Wallis χ^2^ [3] = 12.753; *p* = 0.005), the SM group showed lower weight (in kg) when compared with hypertensive mothers (HM group) (Dunn post hoc test, *p* = 0.028). Additionally, prepregnancy BMI comparisons (Kruskal-Wallis χ^2^ [3] = 17.507; *p* = 0.001) revealed that the CM group showed a lower prepregnancy BMI when compared with the DM and HM groups (Dunn post hoc test, *p* = 0.011 and *p* = 0.019, respectively), and that the SM group showed a lower prepregnancy BMI when compared with the DM and HM groups (Dunn post hoc test, *p* = 0.029 and *p* = 0.032, respectively).

There was a difference in maternal weight preceding the delivery among groups (one-way ANOVA, F [3.120] = 5.472; *p* = 0.001), since the CM and SM groups had a lower weight than the HM group (Tukey post hoc test, *p* = 0.003 and *p* = 0.003, respectively). Maternal weight 15 days postpartum was also different (one-way ANOVA, F [3.120] = 5.857; *p* = 0.001), since the HM group presented a higher weight than the CM and SM groups (Tukey post hoc test, *p* = 0.002 and *p* = 0.003, respectively). There was also a difference in maternal weight 1 month postpartum (one-way ANOVA, F [3.120] = 5.864, *p* = 0.001], considering that the CM and SM groups showed a lower weight when compared with the HM group (Tukey post hoc test, *p* = 0.002 and *p* = 0.003, respectively). Maternal weight and BMI, 6 months postpartum, also differed among the groups (weight: Kruskal-Wallis χ^2^ [3] = 18.121; *p* ≤ 0.0001; BMI: Kruskal-Wallis χ^2^ [3] = 10.334; *p* = 0.016), since the CM and SM groups displayed a lower weight when compared with the HM group (Dunn post hoc test, *p* = 0.002 and *p* = 0.002, respectively), and the HM group showed an increased BMI when compared with the CM group (Dunn post hoc test, *p* = 0.026).

The maternal total calorie intake was retrospectively estimated by the FFQ, which revealed a difference among the groups (Kruskal-Wallis χ^2^ [3] = 10.470, *p* = 0.015), since the DM group consumed fewer calories when compared with the HM group (Dunn post hoc test, *p* = 0.019). Additionally, the present study stratified which macronutrient was consumed in excess: protein calorie intake was not statistically significant among the groups (*p *> 0.05), while carbohydrates and fat were statistically significant (carbohydrates: Kruskal-Wallis χ^2^ [3] = 9.362; *p* = 0.025; fat: Kruskal-Wallis χ^2^ [3] = 8.514,;*p* = 0.036), since the HM group consumed more carbohydrates and fat when compared with the DM group (Dunn post hoc test, carbohydrate *p* = 0.027 and fat *p* = 0.013) [median (95% CI) of carbohydrates: DM = 2270.54 (2155.32 − 2872.71) kcal, HM = 3501.02 (2938.85 − 4154.74) kcal; median (95% CI) of fat: DM = 111.77 (103.40 − 146.02) kcal, HM = 143.21 (134.96 − 216.32)kcal]. The family household income among the studied groups presented a statistically significant difference (Kruskal-Wallis χ^2^ [3] = 13.456, *p* = 0.004), since the SM group had a lower family income when compared with the CM group (Dunn post hoc test, *p* = 0.005). Tables 1 and 2 describe the sample profile and group comparisons in further detail.

**Table 1:** Sample profile—continuous variables

Variables	Total (*n* = 124)	DM (*n* = 32)	HM (*n* = 17)	SM (*n* = 24)	CM (*n* = 51)	*p-value*
Maternal age (years old) median (P25–P75)	29.0(24.0–34.0)	31.0(27.0–35.5)	29.0(27.0–34.0)	28.0(23.5–33.0)	28.0(23.0–33.5)	0.193
Antenatal care visits mean ± SD	8.8 ± 3.0*	9.8 ± 3.1^b^	9.8 ± 1.9^b^	6.9 ± 2.8^a^	8.7 ± 2.9^ab^	0.001
Maternal educational level (in years) median (P25–P75)	10.0(8.0–11.0)*	10.0(6.5–10.0)^b^	11.0(8.0–11.7)^ab^	10.0(7.0–10.3)^b^	11.0(9.5–11.5)^a^	0.037
Maternal height (in m) mean ± SD	1.60 ± 0.06*	1.58 ± 0.07	1.59 ± 0.07	1.59 ± 0.07	1.62 ± 0.06	0.060
Maternal prepregnancy BMI (in kg/m^2^) median (P25–P75)	25.3(21.7–28.8)	27.3(24.7–30.8)^b^	27.4(25.8–32.8)^b^	22.5(19.9–27.6)^a^	23.5(21.2–26.6)^a^	0.001
Maternal calorie intake (in kcal) median (P25–P75)	4,508.8 (3,449.8–6,161.0)	3,687.3 (2,800.1–5,426.9)^a^	5,611.4 (4,492.7–6,824.8)^b^	4,713.6 (3,789.3–8,064.5)^ab^	3,849.6 (3,479.1–5,659.4)^ab^	0.015
PSS-14 score mean ± SD	19.8 ± 8.5*	17.9 ± 6.8	20.1 ± 9.1	20.4 ± 9.9	20.7 ± 8.5	0.548
1^st^-month EPDS score median (P25–P75)	19.0(17.0–20.0)*	20.0(18.0–20.0)	19.0(17.0–20.0)	18.0(15.5–21.0)	19.0(18.0–20.0)	0.873
3^rd^-month EPDS score median (P25–P75)	19.0(17.0–20.0)*	18.5(17.5–20.0)	19.0(17.0–20.0)	18.5(16.0–20.0)	19.0(17.0–20.0)	0.827
6^th^-month EPDS score median (P25–P75)	19.0(17.0–20.0)*	19.0(17.0–20.0)	19.0(18.0–20.0)	18.0(16.0–21.0)	20.0(18.0–20.0)	0.627
Household income^a^	1,712.5 (1,200.0–2,500.0)	1,450.0 (1,150.0–2,000.0)^ab^	1,800.0 (1,000.0–2,400.0)^ab^	1,200.0 (800.0–2,000.0)^b^	2,000.0 (1,470.0–3,000.0)^a^	0.004

Abbreviations: CM, control mothers; DM, diabetic mothers; EPDS, Edinburgh Postpartum Depression Scale; HM, hypertensive mothers; kcal, kilocalories; kg, kilograms; m, meter; P25–P75, 25th and 75th percentiles; PSS, Perceived Stress Scale; SD, standard deviation; SM, smoking mothers.

a Brazilian household income unit reference (2017): BRL 937.00.

a,b Different letters represent statistically different proportions by one-way ANOVA with the Tukey post hoc test or the Kruskal-Wallis test with the Dunn post hoc test.

* Represents statistically different distribution in the total sample by the Chi-squared test. Significance set as *p* ≤ 0.05 for all of the analyzes.

Impact of the Variations of the Perinatal Environment on the Health of the Newborn in the First Six Months of Life (IVAPSA, in the Portuguese acronym) Birth Cohort (*n* = 124), Porto Alegre, state of Rio Grande do Sul, Brazil—September 2011 to January 2016.

**Table 2:** Sample profile—categorical variables

Variables	*n* (*n*%) (*n *= 124)
Maternal skin color	
White	80 (64.5)
Not white	44 (35.5)
Pregnancy planning	
Yes	68 (54.8)
No	56 (45.2)
Marital status	
Married or living with partner	110 (88.7)
Single or living without partner	14 (11.3)
Lactation practice 7 days postpartum	
Exclusive breastfeeding	82 (66.1)
Breastfeeding + other	10 (8.1)
Only other	1 (0.8)
Missing	31 (25.0)
Lactation practice 15 days postpartum	
Exclusive breastfeeding	103 (83.1)
Breastfeeding + other	19 (15.3)
Only other	2 (1.6)
Lactation practice 1 month postpartum	
Exclusive breastfeeding	91 (73.4)
Breastfeeding + other	31 (25.0)
Only other	2 (1.6)
Lactation practice 3 months postpartum	
Exclusive breastfeeding	81 (65.3)
Breastfeeding + other	24 (19.4)
Only other	17 (13.7)
Missing	2 (1.6)
Lactation practice 6 months postpartum	
Exclusive breastfeeding	53 (42.7)
Breastfeeding + other	40 (32.3)
Only other	30 (24.2)
Missing	1 (0.8)
Type of delivery	
Cesarean section	46 (37.1)
Vaginal	78 (62.9)
Received food consumption guidance during gestation	
Yes	78 (62.9)
No	46 (37.1)
Prepregnancy BMI categories	
Normal	55 (44.4)
Underweight	4 (3.2)
Overweight	41 (33.1)
Obese	24 (19.4)
Parity	
0	42 (33.9)
≥ 1	82 (66.1)
Physical activity according to the IPAQ	
Sedentary	9 (7.3)
Irregular active	36 (29.0)
Active	79 (63.7)

Abbreviations: BMI, body mass index; IPAQ, International Physical Activity Questionnaire; n, absolute frequency; n%, relative frequency.

Impact of the Variations of the Perinatal Environment on the Health of the Newborn in the First Six Months of Life (IVAPSA, in the Portuguese acronym) Birth Cohort (*n* = 124), Porto Alegre, state of Rio Grande do Sul, Brazil – September 2011 to January 2016.

**Fig. 1:**
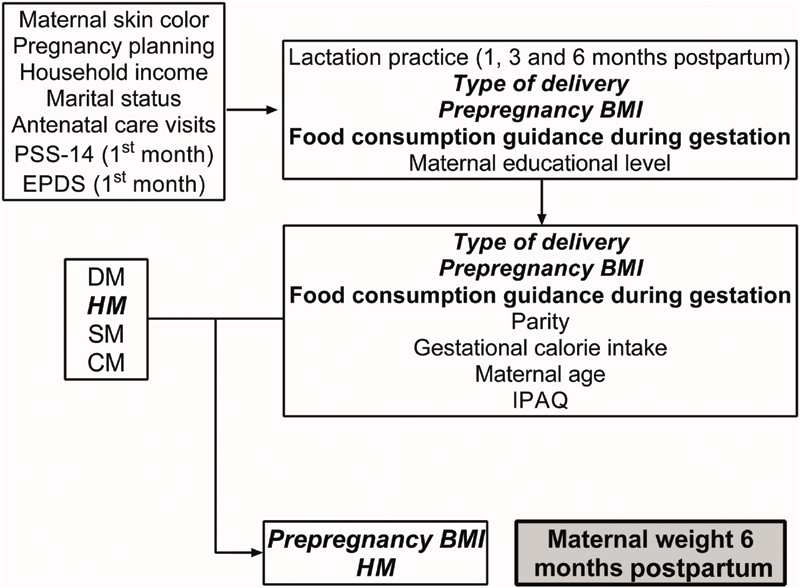
Theoretical framework of risk factors for maternal body weight variation 6 months postpartum structured in hierarchical blocks. Abbreviations: BMI, body mass index; CM, control mothers; DM, diabetic mothers; EPDS, Edinburgh Postnatal Depression Scale; HM, hypertensive mothers; IPAQ, International Physical Activity Questionnaire; PSS-14, 14-item Perceived Stress Scale; SM: smoking mothers.

Regression models for weight variation and associated factors were performed, and the variables less associated with the outcome were excluded. A hierarchical final model was produced by multiple logistic regressions (conducted in four blocks), and it is illustrated in Fig. 1. In summary, the type of delivery, mothers receiving food consumption guidance during the gestation, maternal prepregnancy BMI, and the presence of hypertensive status were correlated with maternal weight gain 6 months postpartum (measured by the difference between the weight measurements at the 6^th^ month of gestation and during prepregnancy). In the proximal block, receiving food consumption guidance during the gestation and type of delivery were excluded, since they were not significantly related to the outcome (*p* ≥ 0.05); in contrast with hypertensive status and overweight prepregnancy BMI, which showed a positive correlation to maternal weight gain 6 months postpartum. All of the other variables were negatively correlated with the outcome.

The GLMs were performed in order to verify how the maternal weight underwent modifications among the groups, among the time measurements, and among the possible groups versus time interactions. For all of the GLMs, the weight measurements were adjusted for maternal height, parity, maternal educational level, type of delivery, and for measurements at 3 different times (prepregnancy, preceding the delivery, and 15 days postpartum). A group (Wald, χ^2^ [3] = 24.748; *p* ≤ 0.0001) and a time (Wald, χ^2^ [5] = 987.100; *p* ≤ 0.0001) effect was observed, without interaction (Wald, χ^2^ [15] = 20.663; *p* = 0.148), since all of the postpartum weights were higher than the measurement taken during prepregnancy. Additionally, the HM and DM groups showed a higher body weight in relation to the CM group 6 months postpartum, but only women in the HM group presented a significant increase in body weight variation, since their weight remained higher from the 15^th^ postpartum day on when compared with their prepregnancy weight. These results are summarized in Table 3.

**Table 3:** Maternal weight modification among groups, measurements and pairwise comparisons

	Prepregnancy	Preceding delivery	15 days postpartum	1 month postpartum	3 months postpartum	6 months postpartum
DM	71.64 ± 12.32^**bcA**^	83.89 ± 12.64^**abB**^	75.43 ± 12.64^**abAC**^	74.97 ± 12.64^**abAC**^	75.49 ± 12.64^**abAC**^	75.64 ± 12.66^**bcAC**^
HM	75.05 ± 12.82^**cA**^	93.10 ± 12.73^**bB**^	83.44 ± 12.66^**bC**^	82.56 ± 12.64^**bC**^	83.12 ± 12.69^**bC**^	84.03 ± 12.65^**cC**^
SM	62.50 ± 12.47^**abA**^	75.97 ± 12.64^**aB**^	67.36 ± 12.57^**aC**^	66.51 ± 12.54^**aD**^	66.44 ± 12.62^**aCD**^	65.84 ± 12.67^**abACD**^
CM	64.29 ± 12.34^**aA**^	77.98 ± 12.35^**aB**^	68.87 ± 12.37^**aC**^	68.11 ± 12.38^**aD**^	68.23 ± 12.39^**aCD**^	67.83 ± 12.38^**aCD**^

Abbreviations: CM, Control mothers; DM, Diabetic mothers; HM, Hypertensive mothers; SM, Smoking mothers.

A group and time effect was observed by maternal weight and measurements (moments) pairwise comparisons by generalized linear models (both *p* ≤ 0.0001). Data expressed as mean ± standard error of mean.

^ab^Different lowercase letters indicate the difference proportion among the studied groups.

^AB^Different uppercase letters show the evolution of a certain group over time. Significance set as *p* ≤ 0.10 for all of the analyzes.

Impact of the Variations of the Perinatal Environment on the Health of the Newborn in the First Six Months of Life (IVAPSA, in the Portuguese acronym) Birth Cohort (*n* = 124), Porto Alegre, state of Rio Grande do Sul, Brazil – September 2011 to January 2016.

## Discussion

In the present research, prepregnancy BMI and hypertensive state influenced the maternal weight variation 6 months postpartum in a sample of different intrauterine environments. The gestation and the postpartum are periods of the reproductive cycle associated with overweight. In the cases of overweight or obesity, the management overweight women requires even more care to avoid the weight outcomes that we found in this study. Our data demonstrated that most of the sample was classified as eutrophic, but the highest prepregnancy BMI values were derived from the HM (27.4kg/m^2^) and DM (27.3kg/m^2^) groups, since excess of weight during prepregnancy favors the development of chronic maternal diseases.^13^

Maternal weight variation can be modulated by several factors, including physical activity and caloric consumption, as well as by receiving dietary guidance. In the present study, although most women received dietary guidance (62.9%), it was supplied from a wide range of professionals and sources, and showed no effect on weight variation 6 months postpartum. These results allow the inference that there was no intervention on maternal dietary practice, only guidance at some point during the antenatal care follow-up. In this context, according to Bye et al,^14^ women with a high BMI are more likely to receive and to follow nutritional counseling compared with those with a normal or below recommended BMI.

Physical activity is another factor that might directly modulate the maternal weight variation 6 months postpartum, although our results showed no significance between physical activity during gestation and the maternal weight variation. Our sample consisted of 63.7% of women who were active during the gestation. Thus, the level of physical activity is an important parameter for the health of our population, especially for overweight and prepregnancy obesity ranges, as other studies have reported.^15^

The calorie intake during gestation was estimated by the FFQ, although it simply estimates the intake of calories and is usually used to elicit a comprehensive estimate of the average diets of the subjects. Despite this limitation, the FFQ is a widely instrument used in epidemiological studies, and our results revealed no influence of this estimative on the maternal weight variation, but the highest calorie intake was observed in the HM group, with a median value estimated at > 5611.4 Kcal/day. Additionally, protein calorie intake was not significant among the groups, while carbohydrates and fat were, since the HM group consumed more carbohydrates and fat when compared with the DM group. These results give us the hypothesis that, in the HM group, the total calorie intake was high. At this point, we emphasize that this is a cohort enriched of different intrauterine environments, and that the adherence to dietary guidelines should be considered more important for the HM group. We also emphasize that all of the women diagnosed with DM underwent, during the pregnancy, a rigorous dietary treatment program in the studied hospitals, which may explain the lower caloric intake of this group in relation to the HM group. A study also demonstrate the importance of assessing calorie intake and, hence, of developing more specific interventions for early pregnancy and postpartum.^16^

Siega-Riz et al^17^ state that few prospective studies with pregnant women and women in the puerperal period analyzed simultaneously the associations of multiple sociodemographic, perinatal, behavioral, and psychosocial factors with postpartum weight in a comprehensive manner, such as the one performed in the present study. It was observed that our sample was predominantly married or reported living with their partners (88.7%). Regarding ethnicity, there was a higher frequency of white women in our study (64.5%). In this perspective, despite the differences in family income and educational level, our sample was very homogeneous. Further studies addressing a more diverse population, based on ethnicity, are needed. Therefore, it is known that cultural and ethnic differences can influence the controversial results reported by some studies.

Considering the socioeconomic level, 113 (91.1%) of the sample had low socioeconomic status (0–2 minimum wages), while in the study reported by Siega-Riz et al,^17^ approximately one quarter of the women were considered as having a low-income status. The Institute of Medicine^18^ affirmed that a low socioeconomic level increases the risk of developing overweight and obesity due to the greater vulnerability to diets with high caloric density and low nutritional value, as well as to the lower practice of physical activity, highlighting the importance of studies on and of management for this population.

The number of antenatal care visits has a protective effect on the health of the pregnant woman and of the newborn, contributing to a reduction in the risk of maternal and infant death, of low birthweight, and of other comorbidities.^18^ Our data demonstrated that the HM and DM groups displayed the highest number of antenatal care visits, being this fact possibly justified by the presence of a chronic disease and the need for better self-care and more attention from health teams.

The transition to motherhood is a period of social, psychological, and behavioral changes in the lives of women. In the present study, psychological variables were also assessed, since many studies reported a relationship between maternal weight variation, depressive symptoms, and stress perception.^20^ Using validated self-administered questionnaires, our study evaluated the maternal presence of depressive symptoms and the level of perceived stress by the mothers, and found no significant difference between depressive symptoms, stress perception, and postpartum maternal weight variation. However, an US cohort study reported that higher levels of stress perception and of depressive symptoms were associated with a higher maternal weight 6 and 12 months postpartum.^19^ In contrast, another US study also reported that a low maternal level of perceived stress is associated with a higher maternal weight 3 months postpartum.^21^

After this initial identification, a hierarchical model was performed in four blocks by multiple linear regressions, distributing the proximal and the distal variables in relation to maternal weight variation 6 months postpartum. Among all of the analyzed variables in the intermediate processes, food consumption guidance was significantly related to the outcome. However, it was evidenced that this effect lost the significance when added to the more proximal variables (that is, prepregnancy BMI, type of delivery, and HM group), of which only BMI and the HM group remained significant until the final model. The food consumption guidance was given by the most diverse professionals and not only by professionals qualified for this, such as nutritionists. In addition, they were made homogeneously between the groups, showing no significant difference.

Considering the type of delivery, according to Bautista-Castaño et al,^22^ the risk for cesarean section was increased for overweight women; nevertheless, this risk may be related to the fact that obesity during pregnancy predisposes to complications related to chronic diseases and fetal macrosomia. Likewise, in comparison to women with adequate BMI, the risk of cesarean delivery increased 50% in the case of overweight women, and this value could double in obesity cases.^23^ In the present study, 33.1% of the women had a prepregnancy BMI classified as overweight. Another observational study performed in Brazil also drew attention to this factor: the large number of women, who began gestation in the overweight and obesity ranges, contributes to a worse outcome in relation to postpartum weight variation.^24^ This data demonstrates the importance that must be given to this population, by means of public policies aiming women in the reproductive age, and not just for the extremes of low weight and obesity.

In the present study, gestational weight gain was not classified. However, maternal weight differences were estimated between all of the points of the curve by multivariable regressions. Thus, nonparametric comparisons and generalized estimates were performed for each time point of maternal weight measurement.

Analyses of variance revealed that the SM and CM groups behaved similarly for all weight measurements, and that the HM group presented a greater body weight in relation to both the SM and the CM groups. It is known that maternal smoking has a negative influence on maternal and child health, mainly due to the effects of nicotine on metabolic parameters. As described in animal and human studies, nicotine use during pregnancy results in anorexia and in increased metabolism, which leads to weight loss, in addition to stimulating the melanocortin 4 receptor, resulting in reduced consumption of food.^25^ This evidence may explain why the women in the SM group behaved similarly to the women in the CM group.

Overweight and obesity are important factors in the establishment of chronic diseases. Especially during the reproductive period, maternal nutritional status predisposes to adverse conditions, as has been demonstrated in studies with populations similar to ours.^26^ Considering both the analyses and the four intrauterine environments, the HM group presented a greater body weight at all points of measurement. In fact, the HM group was the only significant and proximal intrauterine environment related to the outcome in the hierarchical model: these mothers had more weight preceding delivery, which remained greater in all of the time measurements, compared with the other intrauterine environments. The hypertensive status displayed a positive β regression coefficient to the outcome, since belonging to the HM group already attributed ∼ 5 kg to the maternal weight variation 6 months postpartum.

In addition, underweight and obesity prepregnancy BMI were negatively correlated with the outcome. On the other hand, women classified as overweight presented results that were similar to the effects of hypertension. In our analysis, the HM group was represented mainly by arterial hypertension during pregnancy (45.9%), and pre-eclampsia (29.7%). Hypertensive disorders affect ∼ 10% of the pregnancies,^27^ and represent one of the main causes of maternal death, associated with high rates of perinatal morbidity and mortality.^28^ Some studies have shown that overweight is an important risk factor for the development of hypertensive disorders during pregnancy.^29^ Thadhani et al^30^ evaluated the relationship between prepregnancy BMI, cholesterol levels, and the risk of developing gestational hypertensive disorders: they concluded that the higher the prepregnancy BMI, the greater the risk of developing hypertension. These findings may explain our results, since the HM group demonstrated a higher prepregnancy BMI, as well as higher postpartum weight, in relation to the CM group. It is known that hypertensive disorders during gestation predispose mothers to future cardiovascular diseases,^31^ and this is an important fact to be considered when analyzing this population.

## Strengths and Limitations

Although the present study demonstrated that underweight and obese prepregnancy BMIs were negatively correlated to the outcome, and that hypertension status and overweight prepregnancy BMI were positively correlated to the outcome, certain limitations should be considered when evaluating these results. First, the present study represents only a part of the sample of the Impact of the Variations of the Perinatal Environment on the Health of the Newborn in the First Six Months of Life (IVAPSA, in the Portuguese acronym) birth cohort. It is believed that some of the findings failed to reach statistical significance because the sample size was relatively small for some comparisons (type II error). Additionally, there is the possibility that one or more significant findings may reflect a type I error. Second, the current sample is rather homogeneous. Future research should also examine maternal weight variation and associated factors in samples that are more heterogeneous in terms of their sociodemographic characteristics. Third, the present study used reported data collected 6 months postpartum to provide information about factors associated to maternal weight variation. Longer investigations, or investigations with more repetitions of these measurements are needed. It is important to note that our study is limited by the oversampling of different intrauterine environments, which may restrict our external validity. The extent to which hypertension and overweight prepregnancy BMI status is representative to maternal weight variation remains to be investigated. In addition, although there are studies relating micronutrient supplementation with perinatal outcomes (such as folic acid and iron), the present study did not analyze the influence of the consumption of micronutrients on maternal body weight. More studies are needed to elucidate the possible mechanisms by which these variables influence the vulnerability to maternal weight retention, especially during the puerperal period. Despite all these limitations, this research offered minimal risks to the participants, as well as to the researchers, by employing techniques and methods of data collection in which no intentional intervention or modification were made in the physiological, psychological, and social variables of the subjects (application of questionnaires and review of medical files). In addition, data collection was performed through common procedures in routine physical examinations, such as weight and length measurements. With the present research, it was possible to improve the knowledge about the influence of different intrauterine environments on maternal health to improve intervention strategies for the promotion of health during antenatal care.

## Conclusion

In summary, there are many studies that assess maternal weight variation and its associated factors in the most varied outcomes. Additionally, the thematic of different intrauterine environments is also explored, but usually highlighting its implications in the development of the offspring. Our study aimed to identify the influence of these environments in maternal health, which also affects the health of the newborn. Despite many reported results, which increased the knowledge about the etiological and clinical aspects of gestational hypertensive disorders, the incidence of these disorders is not decreasing, and they are the main cause of maternal mortality and of adverse effects in low- and middle-income countries. In this context, it can be concluded that the BMI of women in the reproductive age is the first warning sign for an intervention in their lifestyle, mainly aiming to reduce possible morbidities resulting from unfavorable nutritional status during pregnancy.
